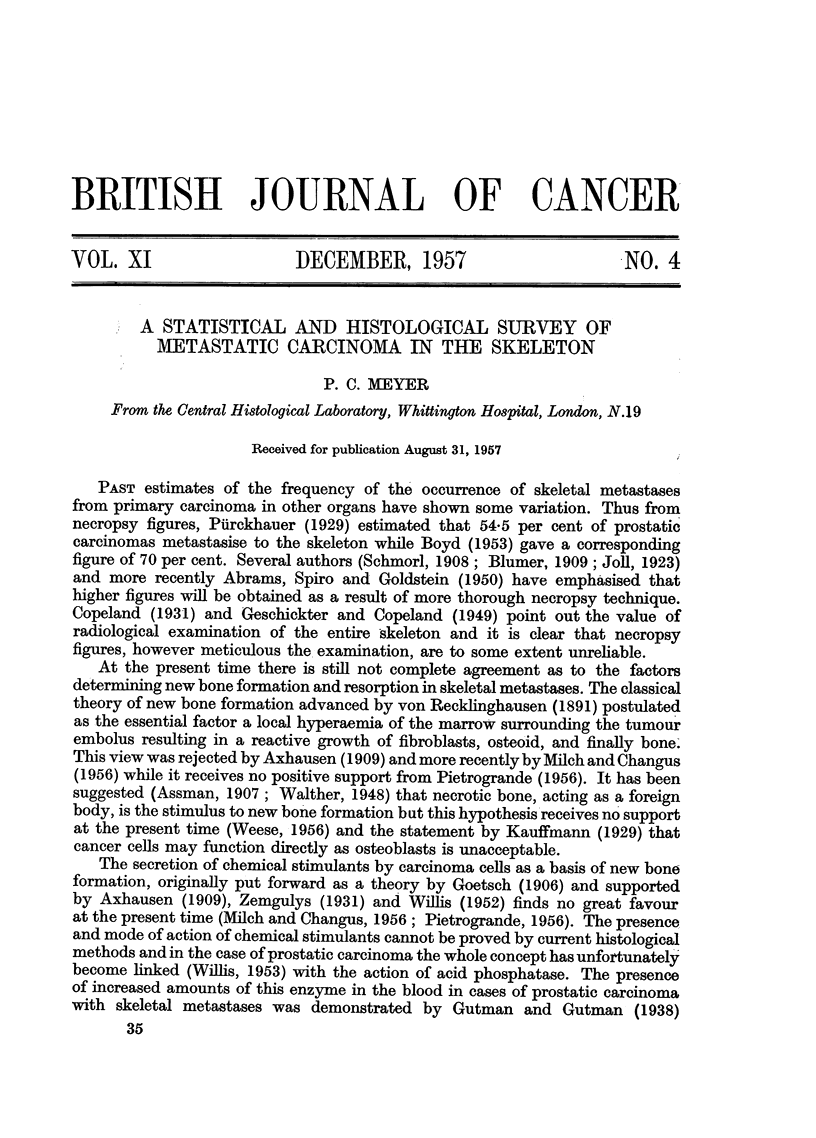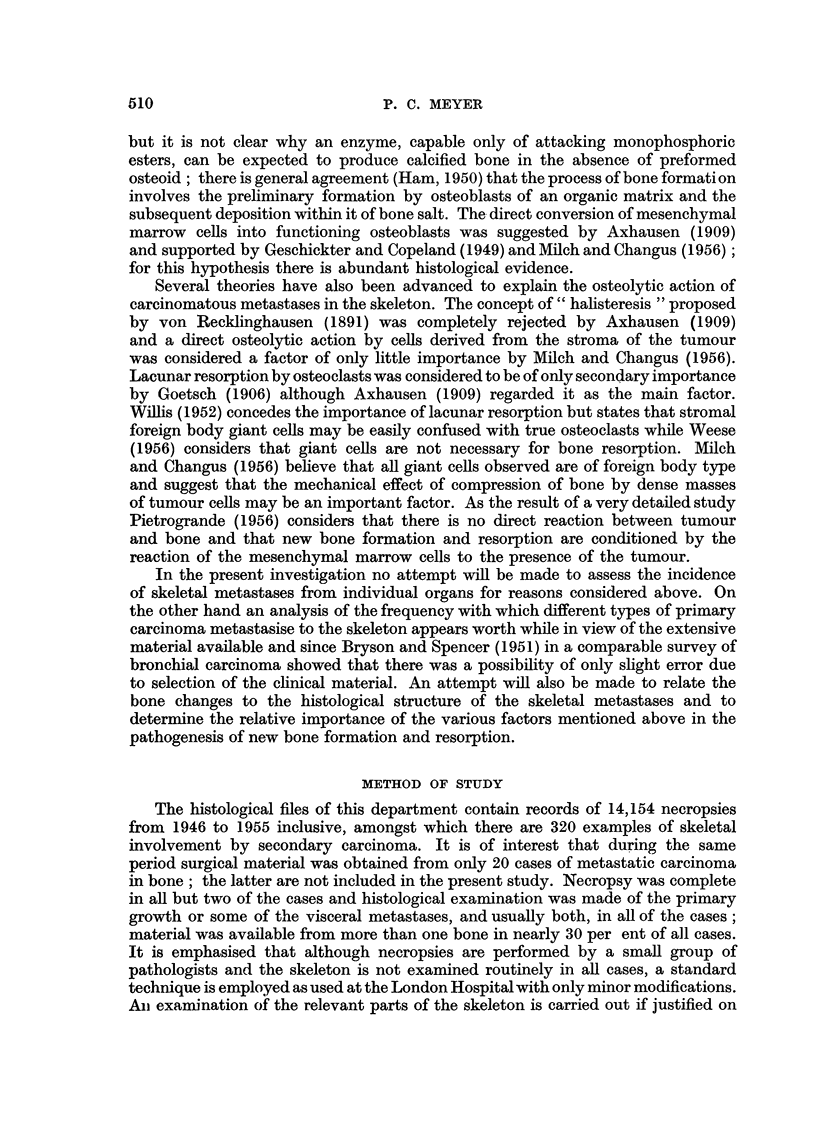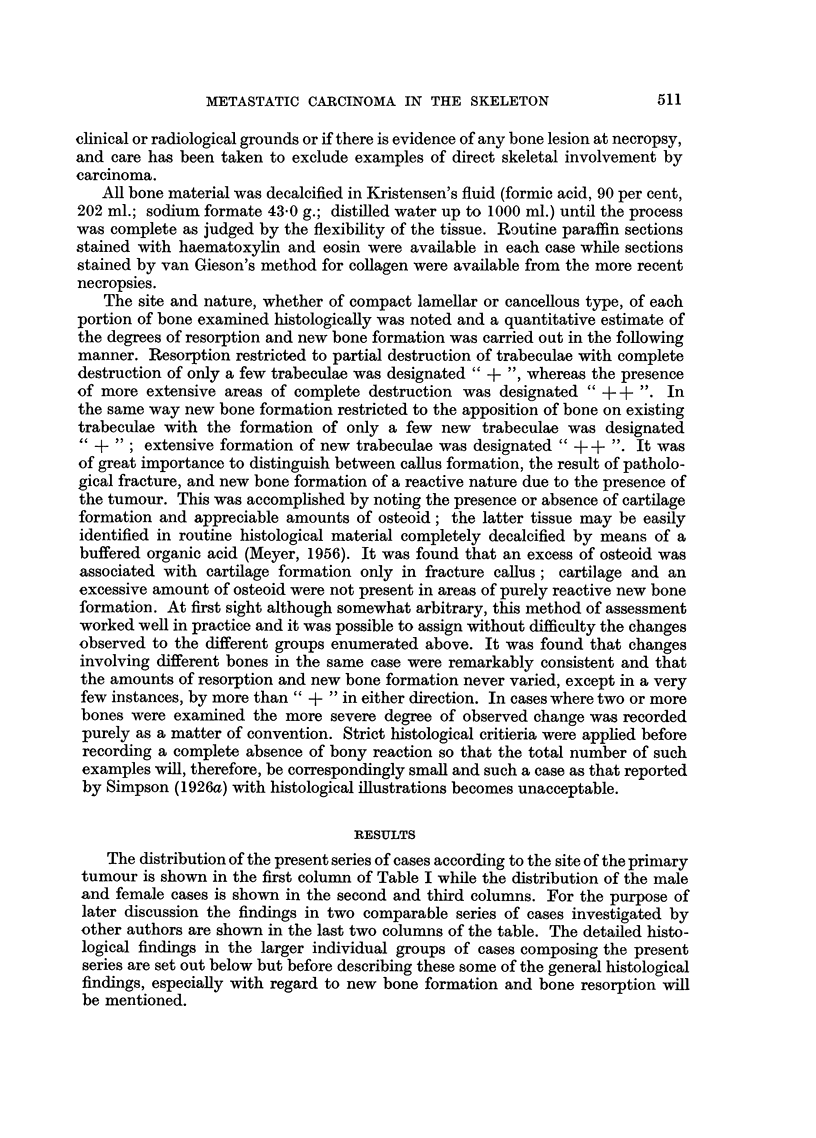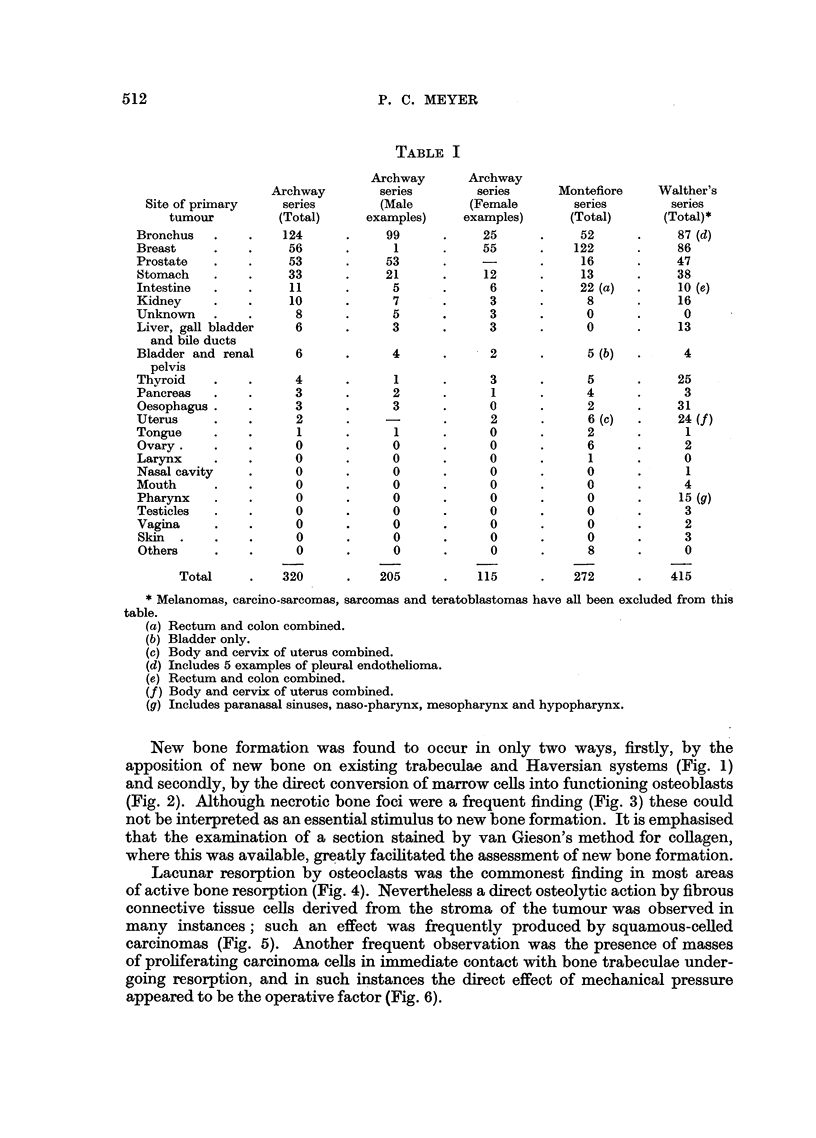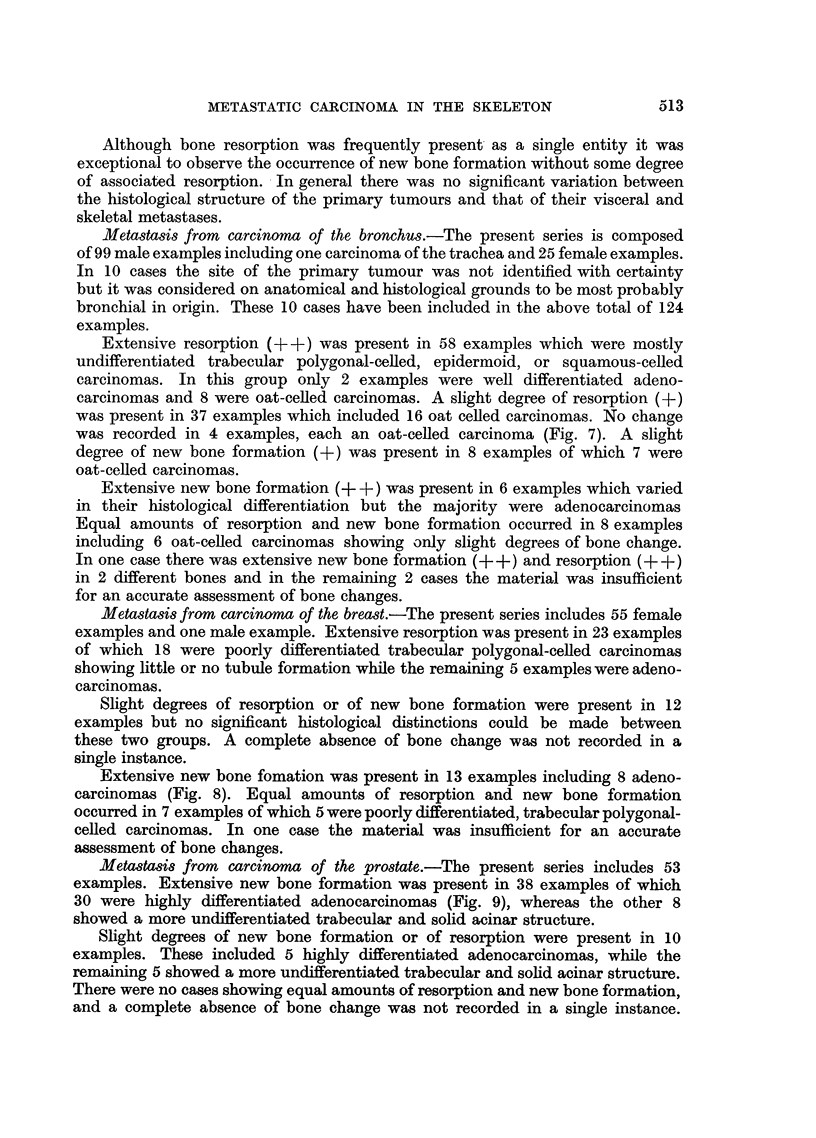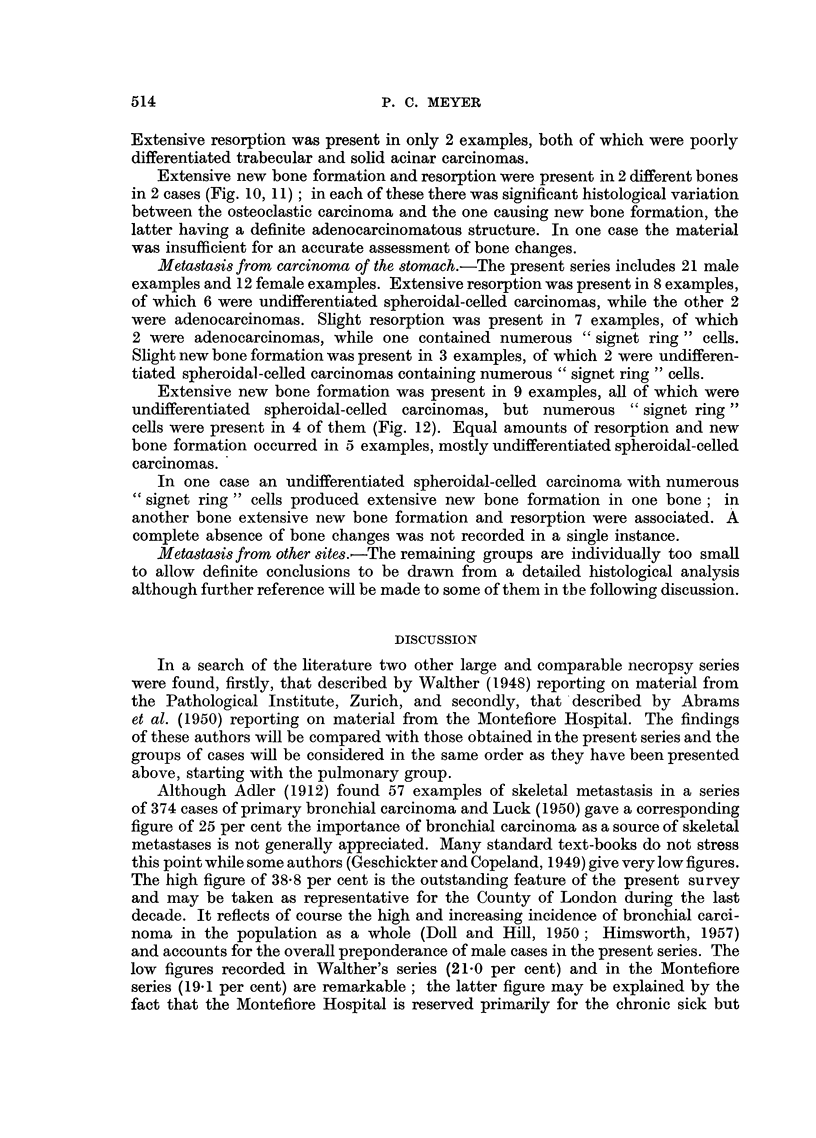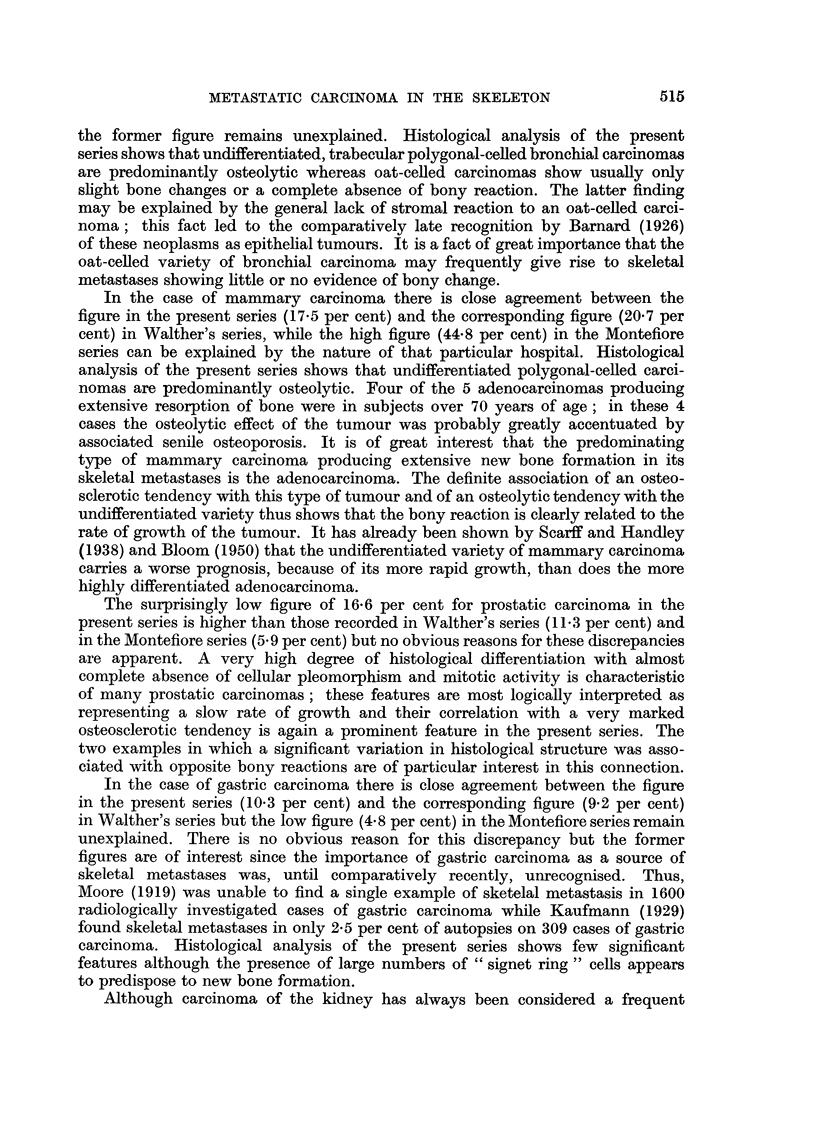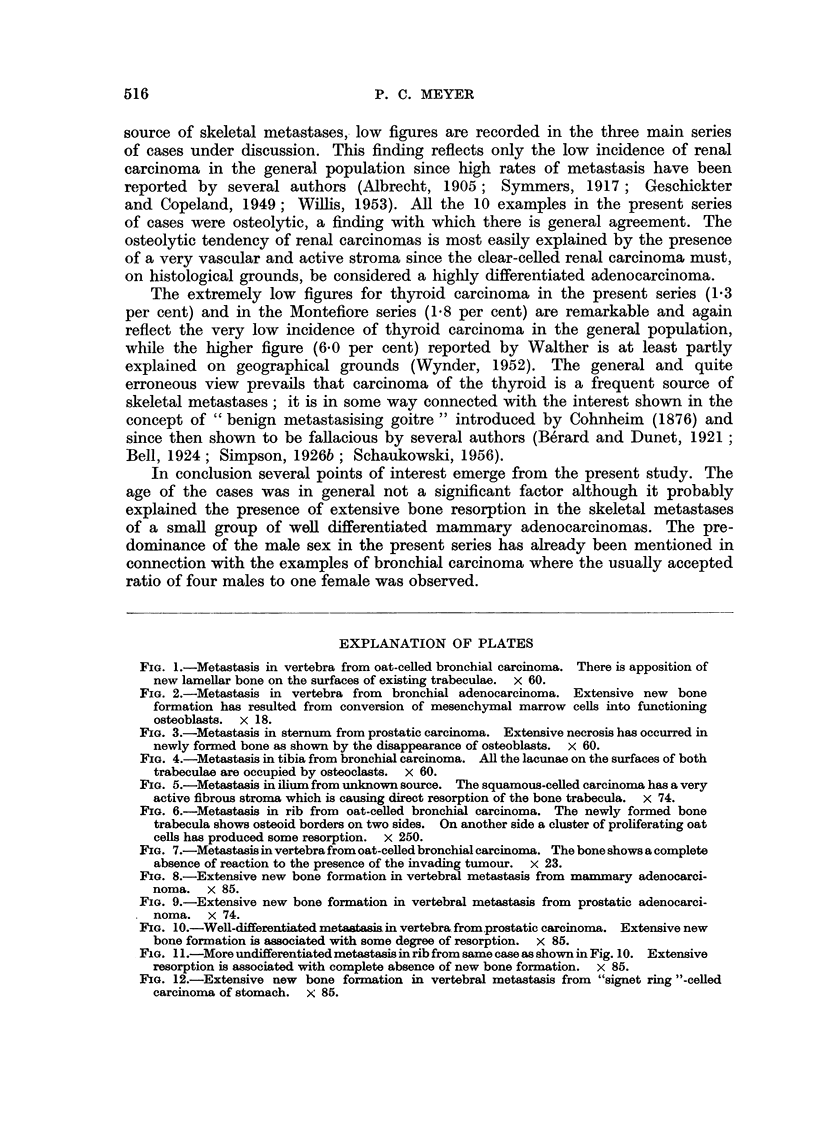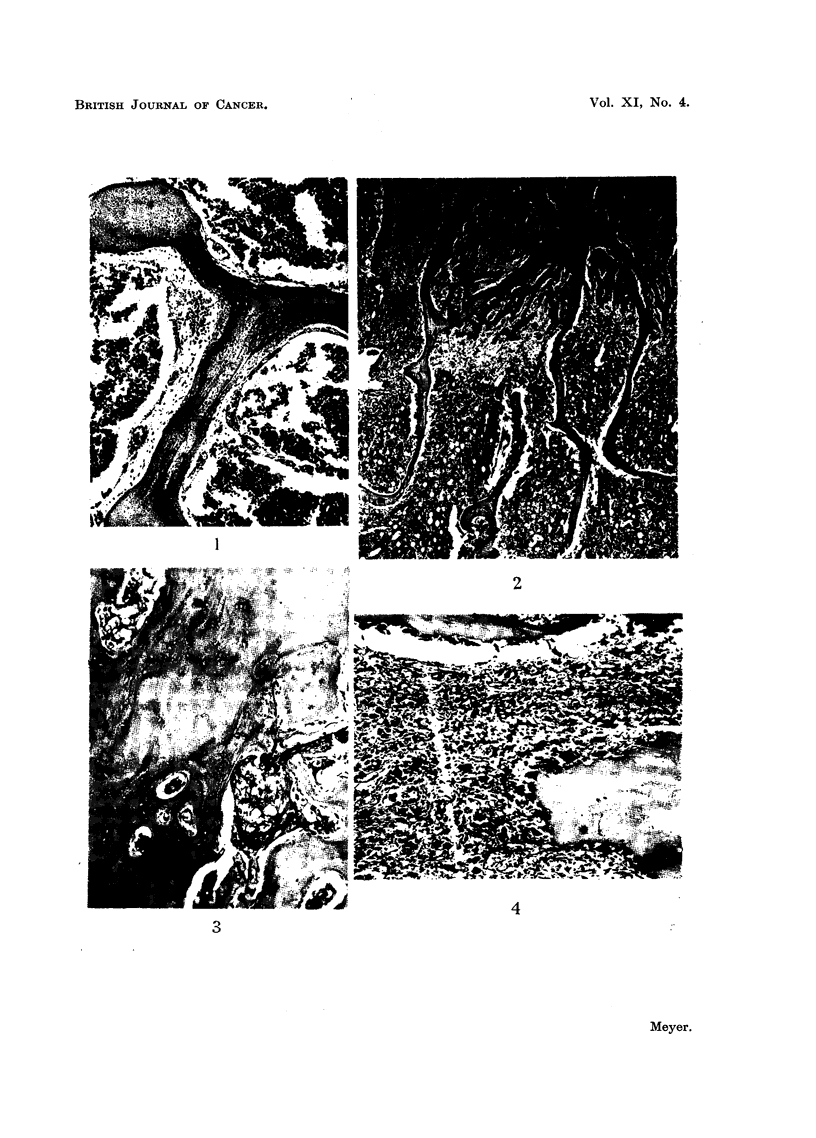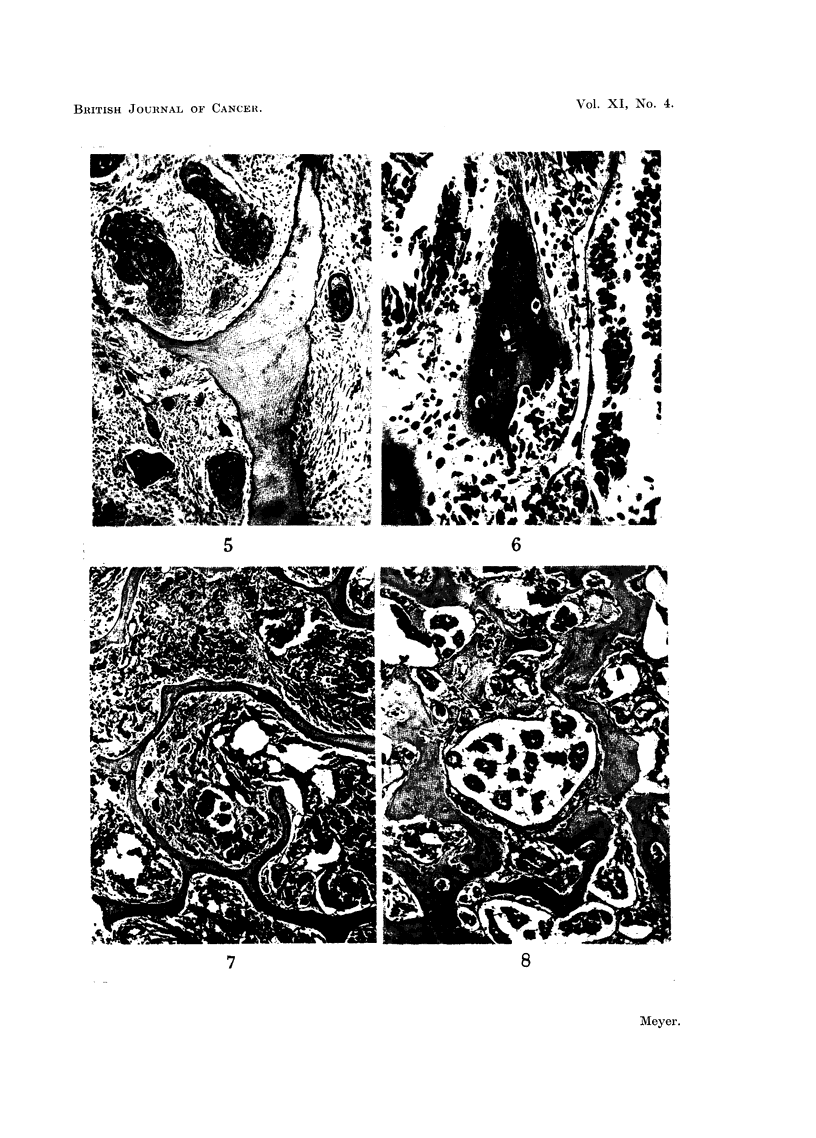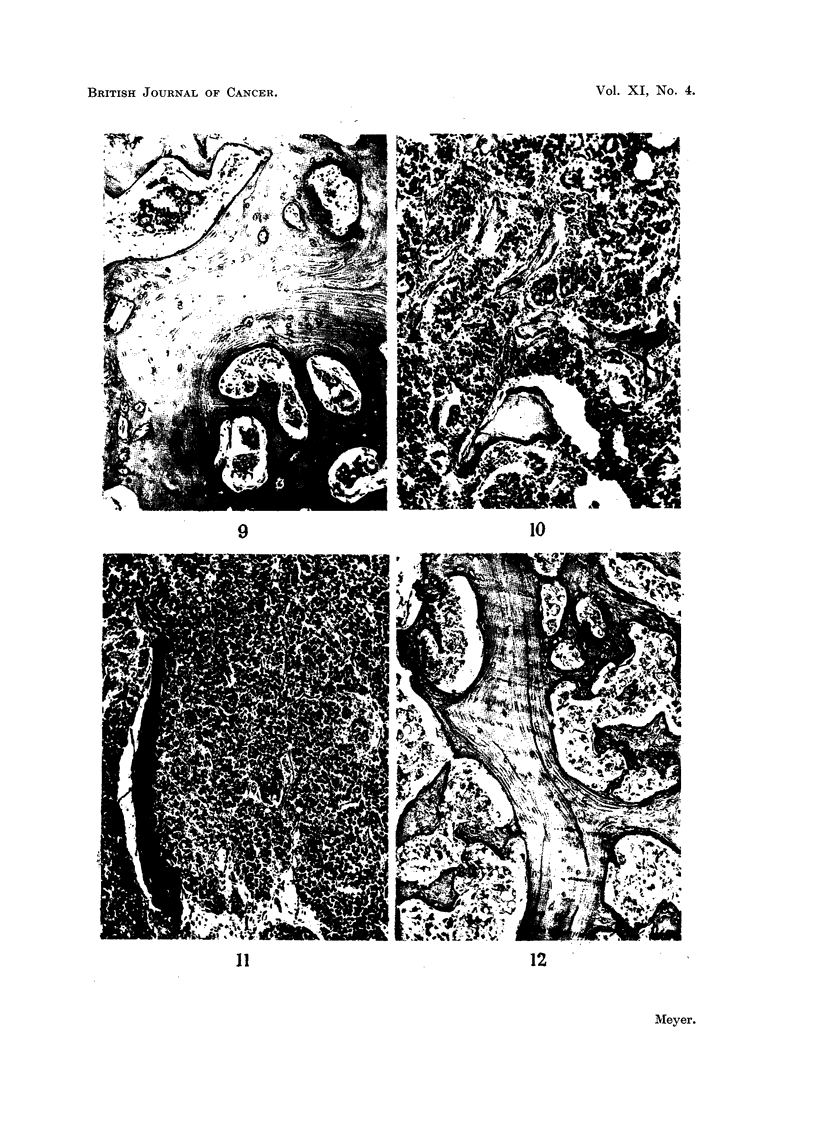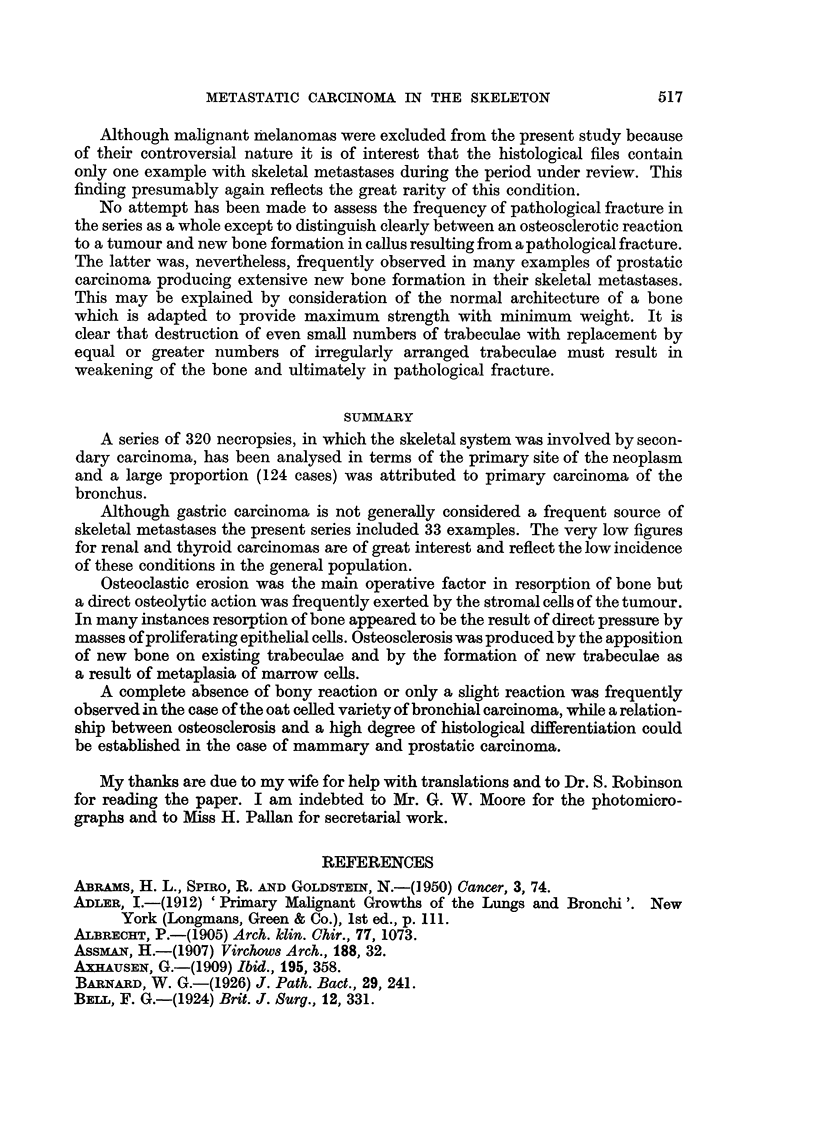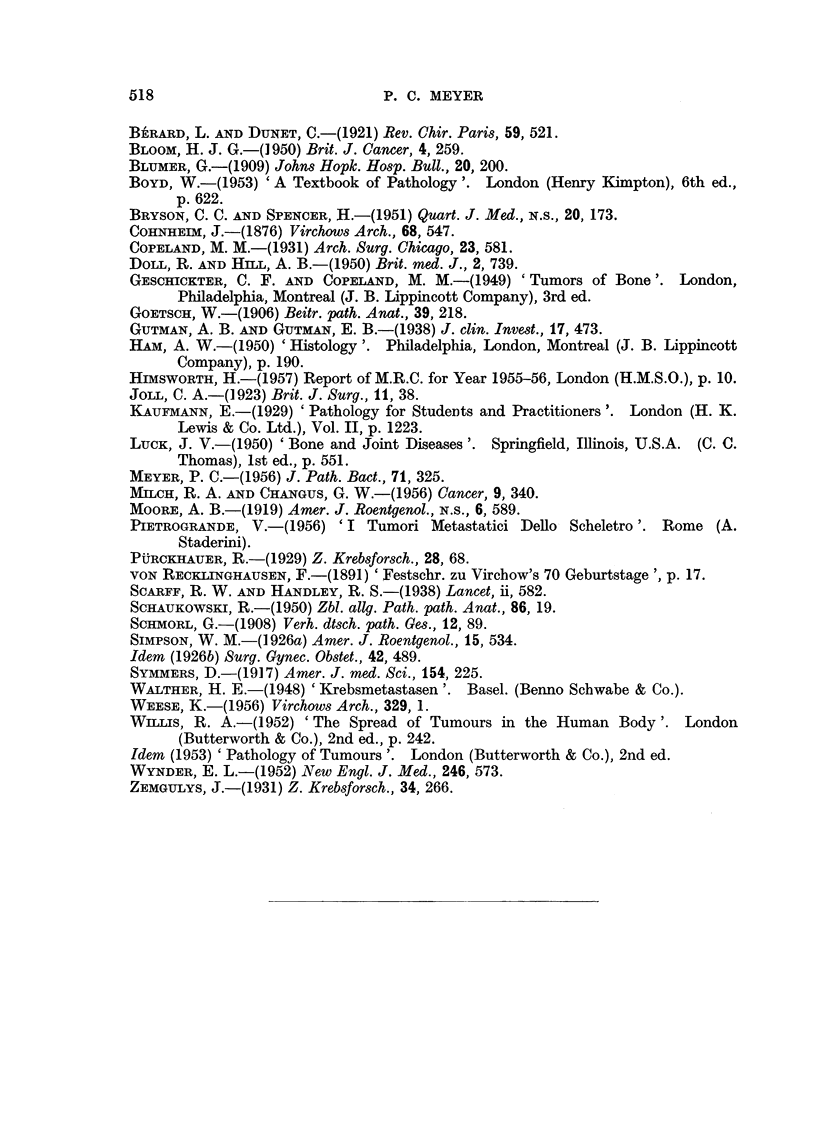# A Statistical and Histological Survey of Metastatic Carcinoma in the Skeleton

**DOI:** 10.1038/bjc.1957.62

**Published:** 1957-12

**Authors:** P. C. Meyer

## Abstract

**Images:**


BRITISH JOURNAL OF CANCER

VOL. XI DECEMBER, 1957 NO. 4

### A STATISTICAL AND HISTOLOGICAL SURVEY OF METASTATIC CARCINOMA IN THE SKELETON

P. C. MEYER

*From the Central Histological Laboratory, Whittington Hospital, London, N.*19

Received for publication August 31, 1957

PAST estimates of the frequency of the occurrence of skeletal metastases from primary carcinoma in other organs have shown some variation. Thus from necropsy figures, Pürckhauer (1929) estimated that 54·5 per cent of prostatic carcinomas metastasise to the skeleton while Boyd (1953) gave a corresponding figure of 70 per cent. Several authors (Schmorl, 1908 ; Blumer, 1909 ; Joll, 1923) and more recently Abrams, Spiro and Goldstein (1950) have emphasised that higher figures will be obtained as a result of more thorough necropsy technique. Copeland (1931) and Geschickter and Copeland (1949) point out the value of radiological examination of the entire skeleton and it is clear that necropsy figures, however meticulous the examination, are to some extent unreliable.

At the present time there is still not complete agreement as to the factors determining new bone formation and resorption in skeletal metastases. The classical theory of new bone formation advanced by von Recklinghausen (1891) postulated as the essential factor a local hyperaemia of the marrow surrounding the tumour embolus resulting in a reactive growth of fibroblasts, osteoid, and finally bone. This view was rejected by Axhausen (1909) and more recently by Milch and Changus (1956) while it receives no positive support from Pietrogrande (1956). It has been suggested (Assman, 1907 ; Walther, 1948) that necrotic bone, acting as a foreign body, is the stimulus to new bone formation but this hypothesis receives no support at the present time (Weese, 1956) and the statement by Kauffmann (1929) that cancer cells may function directly as osteoblasts is unacceptable.

The secretion of chemical stimulants by carcinoma cells as a basis of new bone formation, originally put forward as a theory by Goetsch (1906) and supported by Axhausen (1909), Zemgulys (1931) and Willis (1952) finds no great favour at the present time (Milch and Changus, 1956 ; Pietrogrande, 1956). The presence and mode of action of chemical stimulants cannot be proved by current histological methods and in the case of prostatic carcinoma the whole concept has unfortunately become linked (Willis, 1953) with the action of acid phosphatase. The presence of increased amounts of this enzyme in the blood in cases of prostatic carcinoma with skeletal metastases was demonstrated by Gutman and Gutman (1938)

but it is not clear why an enzyme, capable only of attacking monophosphoric esters, can be expected to produce calcified bone in the absence of preformed osteoid ; there is general agreement (Ham, 1950) that the process of bone formation involves the preliminary formation by osteoblasts of an organic matrix and the subsequent deposition within it of bone salt. The direct conversion of mesenchymal marrow cells into functioning osteoblasts was suggested by Axhausen (1909) and supported by Geschickter and Copeland (1949) and Milch and Changus (1956) ; for this hypothesis there is abundant histological evidence.

Several theories have also been advanced to explain the osteolytic action of carcinomatous metastases in the skeleton. The concept of " halisteresis " proposed by von Recklinghausen (1891) was completely rejected by Axhausen (1909) and a direct osteolytic action by cells derived from the stroma of the tumour was considered a factor of only little importance by Milch and Changus (1956). Lacunar resorption by osteoclasts was considered to be of only secondary importance by Goetsch (1906) although Axhausen (1909) regarded it as the main factor. Willis (1952) concedes the importance of lacunar resorption but states that stromal foreign body giant cells may be easily confused with true osteoclasts while Weese (1956) considers that giant cells are not necessary for bone resorption. Milch and Changus (1956) believe that all giant cells observed are of foreign body type and suggest that the mechanical effect of compression of bone by dense masses of tumour cells may be an important factor. As the result of a very detailed study Pietrogrande (1956) considers that there is no direct reaction between tumour and bone and that new bone formation and resorption are conditioned by the reaction of the mesenchymal marrow cells to the presence of the tumour.

In the present investigation no attempt will be made to assess the incidence of skeletal metastases from individual organs for reasons considered above. On the other hand an analysis of the frequency with which different types of primary carcinoma metastasise to the skeleton appears worth while in view of the extensive material available and since Bryson and Spencer (1951) in a comparable survey of bronchial carcinoma showed that there was a possibility of only slight error due to selection of the clinical material. An attempt will also be made to relate the bone changes to the histological structure of the skeletal metastases and to determine the relative importance of the various factors mentioned above in the pathogenesis of new bone formation and resorption.

### METHOD OF STUDY

The histological files of this department contain records of 14,154 necropsies from 1946 to 1955 inclusive, amongst which there are 320 examples of skeletal involvement by secondary carcinoma. It is of interest that during the same period surgical material was obtained from only 20 cases of metastatic carcinoma in bone ; the latter are not included in the present study. Necropsy was complete in all but two of the cases and histological examination was made of the primary growth or some of the visceral metastases, and usually both, in all of the cases ; material was available from more than one bone in nearly 30 per ent of all cases. It is emphasised that although necropsies are performed by a small group of pathologists and the skeleton is not examined routinely in all cases, a standard technique is employed as used at the London Hospital with only minor modifications. An examination of the relevant parts of the skeleton is carried out if justified on clinical or radiological grounds or if there is evidence of any bone lesion at necropsy, and care has been taken to exclude examples of direct skeletal involvement by carcinoma.

All bone material was decalcified in Kristensen's fluid (formic acid, 90 per cent, 202 ml.; sodium formate 43·0 g.; distilled water up to 1000 ml.) until the process was complete as judged by the flexibility of the tissue. Routine paraffin sections stained with haematoxylin and eosin were available in each case while sections stained by van Gieson's method for collagen were available from the more recent necropsies.

The site and nature, whether of compact lamellar or cancellous type, of each portion of bone examined histologically was noted and a quantitative estimate of the degrees of resorption and new bone formation was carried out in the following manner. Resorption restricted to partial destruction of trabeculae with complete destruction of only a few trabeculae was designated " + ", whereas the presence of more extensive areas of complete destruction was designated " ++ ". In the same way new bone formation restricted to the apposition of bone on existing trabeculae with the formation of only a few new trabeculae was designated " + " ; extensive formation of new trabeculae was designated " ++ ". It was of great importance to distinguish between callus formation, the result of pathological fracture, and new bone formation of a reactive nature due to the presence of the tumour. This was accomplished by noting the presence or absence of cartilage formation and appreciable amounts of osteoid ; the latter tissue may be easily identified in routine histological material completely decalcified by means of a buffered organic acid (Meyer, 1956). It was found that an excess of osteoid was associated with cartilage formation only in fracture callus ; cartilage and an excessive amount of osteoid were not present in areas of purely reactive new bone formation. At first sight although somewhat arbitrary, this method of assessment worked well in practice and it was possible to assign without difficulty the changes observed to the different groups enumerated above. It was found that changes involving different bones in the same case were remarkably consistent and that the amounts of resorption and new bone formation never varied, except in a very few instances, by more than " + " in either direction. In cases where two or more bones were examined the more severe degree of observed change was recorded purely as a matter of convention. Strict histological critieria were applied before recording a complete absence of bony reaction so that the total number of such examples will, therefore, be correspondingly small and such a case as that reported by Simpson (1926*a*) with histological illustrations becomes unacceptable.

### RESULTS

The distribution of the present series of cases according to the site of the primary tumour is shown in the first column of Table I while the distribution of the male and female cases is shown in the second and third columns. For the purpose of later discussion the findings in two comparable series of cases investigated by other authors are shown in the last two columns of the table. The detailed histological findings in the larger individual groups of cases composing the present series are set out below but before describing these some of the general histological findings, especially with regard to new bone formation and bone resorption will be mentioned.

TABLE I

| Site of primary tumour | Archway series (Total) | Archway series (Male examples) | Archway series (Female examples) | Montefiore series (Total) | Walther's series (Total)* |
|---|---|---|---|---|---|
| Bronchus | 124 | 99 | 25 | 52 | 87 (*d*) |
| Breast | 56 | 1 | 55 | 122 | 86 |
| Prostate | 53 | 53 | — | 16 | 47 |
| Stomach | 33 | 21 | 12 | 13 | 38 |
| Intestine | 11 | 5 | 6 | 22 (*a*) | 10 (*e*) |
| Kidney | 10 | 7 | 3 | 8 | 16 |
| Unknown | 8 | 5 | 3 | 0 | 0 |
| Liver, gall bladder and bile ducts | 6 | 3 | 3 | 0 | 13 |
| Bladder and renal pelvis | 6 | 4 | 2 | 5 (*b*) | 4 |
| Thyroid | 4 | 1 | 3 | 5 | 25 |
| Pancreas | 3 | 2 | 1 | 4 | 3 |
| Oesophagus | 3 | 3 | 0 | 2 | 31 |
| Uterus | 2 | — | 2 | 6 (*c*) | 24 (*f*) |
| Tongue | 1 | 1 | 0 | 2 | 1 |
| Ovary | 0 | 0 | 0 | 6 | 2 |
| Larynx | 0 | 0 | 0 | 1 | 0 |
| Nasal cavity | 0 | 0 | 0 | 0 | 1 |
| Mouth | 0 | 0 | 0 | 0 | 4 |
| Pharynx | 0 | 0 | 0 | 0 | 15 (*g*) |
| Testicles | 0 | 0 | 0 | 0 | 3 |
| Vagina | 0 | 0 | 0 | 0 | 2 |
| Skin | 0 | 0 | 0 | 0 | 3 |
| Others | 0 | 0 | 0 | 8 | 0 |
| Total | 320 | 205 | 115 | 272 | 415 |

* Melanomas, carcino-sarcomas, sarcomas and teratoblastomas have all been excluded from this table.
(*a*) Rectum and colon combined.
(*b*) Bladder only.
(*c*) Body and cervix of uterus combined.
(*d*) Includes 5 examples of pleural endothelioma.
(*e*) Rectum and colon combined.
(*f*) Body and cervix of uterus combined.
(*g*) Includes paranasal sinuses, naso-pharynx, mesopharynx and hypopharynx.

New bone formation was found to occur in only two ways, firstly, by the apposition of new bone on existing trabeculae and Haversian systems (Fig. 1) and secondly, by the direct conversion of marrow cells into functioning osteoblasts (Fig. 2). Although necrotic bone foci were a frequent finding (Fig. 3) these could not be interpreted as an essential stimulus to new bone formation. It is emphasised that the examination of a section stained by van Gieson's method for collagen, where this was available, greatly facilitated the assessment of new bone formation.

Lacunar resorption by osteoclasts was the commonest finding in most areas of active bone resorption (Fig. 4). Nevertheless a direct osteolytic action by fibrous connective tissue cells derived from the stroma of the tumour was observed in many instances; such an effect was frequently produced by squamous-celled carcinomas (Fig. 5). Another frequent observation was the presence of masses of proliferating carcinoma cells in immediate contact with bone trabeculae undergoing resorption, and in such instances the direct effect of mechanical pressure appeared to be the operative factor (Fig. 6).

Although bone resorption was frequently present as a single entity it was exceptional to observe the occurrence of new bone formation without some degree of associated resorption. In general there was no significant variation between the histological structure of the primary tumours and that of their visceral and skeletal metastases.

*Metastasis from carcinoma of the bronchus.*—The present series is composed of 99 male examples including one carcinoma of the trachea and 25 female examples. In 10 cases the site of the primary tumour was not identified with certainty but it was considered on anatomical and histological grounds to be most probably bronchial in origin. These 10 cases have been included in the above total of 124 examples.

Extensive resorption (++) was present in 58 examples which were mostly undifferentiated trabecular polygonal-celled, epidermoid, or squamous-celled carcinomas. In this group only 2 examples were well differentiated adenocarcinomas and 8 were oat-celled carcinomas. A slight degree of resorption (+) was present in 37 examples which included 16 oat celled carcinomas. No change was recorded in 4 examples, each an oat-celled carcinoma (Fig. 7). A slight degree of new bone formation (+) was present in 8 examples of which 7 were oat-celled carcinomas.

Extensive new bone formation (++) was present in 6 examples which varied in their histological differentiation but the majority were adenocarcinomas Equal amounts of resorption and new bone formation occurred in 8 examples including 6 oat-celled carcinomas showing only slight degrees of bone change. In one case there was extensive new bone formation (++) and resorption (++) in 2 different bones and in the remaining 2 cases the material was insufficient for an accurate assessment of bone changes.

*Metastasis from carcinoma of the breast.*—The present series includes 55 female examples and one male example. Extensive resorption was present in 23 examples of which 18 were poorly differentiated trabecular polygonal-celled carcinomas showing little or no tubule formation while the remaining 5 examples were adenocarcinomas.

Slight degrees of resorption or of new bone formation were present in 12 examples but no significant histological distinctions could be made between these two groups. A complete absence of bone change was not recorded in a single instance.

Extensive new bone fomation was present in 13 examples including 8 adenocarcinomas (Fig. 8). Equal amounts of resorption and new bone formation occurred in 7 examples of which 5 were poorly differentiated, trabecular polygonal-celled carcinomas. In one case the material was insufficient for an accurate assessment of bone changes.

*Metastasis from carcinoma of the prostate.*—The present series includes 53 examples. Extensive new bone formation was present in 38 examples of which 30 were highly differentiated adenocarcinomas (Fig. 9), whereas the other 8 showed a more undifferentiated trabecular and solid acinar structure.

Slight degrees of new bone formation or of resorption were present in 10 examples. These included 5 highly differentiated adenocarcinomas, while the remaining 5 showed a more undifferentiated trabecular and solid acinar structure. There were no cases showing equal amounts of resorption and new bone formation, and a complete absence of bone change was not recorded in a single instance.

Extensive resorption was present in only 2 examples, both of which were poorly differentiated trabecular and solid acinar carcinomas.

Extensive new bone formation and resorption were present in 2 different bones in 2 cases (Fig. 10, 11) ; in each of these there was significant histological variation between the osteoclastic carcinoma and the one causing new bone formation, the latter having a definite adenocarcinomatous structure. In one case the material was insufficient for an accurate assessment of bone changes.

*Metastasis from carcinoma of the stomach.*—The present series includes 21 male examples and 12 female examples. Extensive resorption was present in 8 examples, of which 6 were undifferentiated spheroidal-celled carcinomas, while the other 2 were adenocarcinomas. Slight resorption was present in 7 examples, of which 2 were adenocarcinomas, while one contained numerous " signet ring " cells. Slight new bone formation was present in 3 examples, of which 2 were undifferentiated spheroidal-celled carcinomas containing numerous " signet ring " cells.

Extensive new bone formation was present in 9 examples, all of which were undifferentiated spheroidal-celled carcinomas, but numerous " signet ring " cells were present in 4 of them (Fig. 12). Equal amounts of resorption and new bone formation occurred in 5 examples, mostly undifferentiated spheroidal-celled carcinomas.

In one case an undifferentiated spheroidal-celled carcinoma with numerous " signet ring " cells produced extensive new bone formation in one bone ; in another bone extensive new bone formation and resorption were associated. A complete absence of bone changes was not recorded in a single instance.

*Metastasis from other sites.*—The remaining groups are individually too small to allow definite conclusions to be drawn from a detailed histological analysis although further reference will be made to some of them in the following discussion.

### DISCUSSION

In a search of the literature two other large and comparable necropsy series were found, firstly, that described by Walther (1948) reporting on material from the Pathological Institute, Zurich, and secondly, that described by Abrams *et al.* (1950) reporting on material from the Montefiore Hospital. The findings of these authors will be compared with those obtained in the present series and the groups of cases will be considered in the same order as they have been presented above, starting with the pulmonary group.

Although Adler (1912) found 57 examples of skeletal metastasis in a series of 374 cases of primary bronchial carcinoma and Luck (1950) gave a corresponding figure of 25 per cent the importance of bronchial carcinoma as a source of skeletal metastases is not generally appreciated. Many standard text-books do not stress this point while some authors (Geschickter and Copeland, 1949) give very low figures. The high figure of 38·8 per cent is the outstanding feature of the present survey and may be taken as representative for the County of London during the last decade. It reflects of course the high and increasing incidence of bronchial carcinoma in the population as a whole (Doll and Hill, 1950 ; Himsworth, 1957) and accounts for the overall preponderance of male cases in the present series. The low figures recorded in Walther's series (21·0 per cent) and in the Montefiore series (19·1 per cent) are remarkable ; the latter figure may be explained by the fact that the Montefiore Hospital is reserved primarily for the chronic sick but the former figure remains unexplained. Histological analysis of the present series shows that undifferentiated, trabecular polygonal-celled bronchial carcinomas are predominantly osteolytic whereas oat-celled carcinomas show usually only slight bone changes or a complete absence of bony reaction. The latter finding may be explained by the general lack of stromal reaction to an oat-celled carcinoma; this fact led to the comparatively late recognition by Barnard (1926) of these neoplasms as epithelial tumours. It is a fact of great importance that the oat-celled variety of bronchial carcinoma may frequently give rise to skeletal metastases showing little or no evidence of bony change.

In the case of mammary carcinoma there is close agreement between the figure in the present series (17·5 per cent) and the corresponding figure (20·7 per cent) in Walther's series, while the high figure (44·8 per cent) in the Montefiore series can be explained by the nature of that particular hospital. Histological analysis of the present series shows that undifferentiated polygonal-celled carcinomas are predominantly osteolytic. Four of the 5 adenocarcinomas producing extensive resorption of bone were in subjects over 70 years of age; in these 4 cases the osteolytic effect of the tumour was probably greatly accentuated by associated senile osteoporosis. It is of great interest that the predominating type of mammary carcinoma producing extensive new bone formation in its skeletal metastases is the adenocarcinoma. The definite association of an osteosclerotic tendency with this type of tumour and of an osteolytic tendency with the undifferentiated variety thus shows that the bony reaction is clearly related to the rate of growth of the tumour. It has already been shown by Scarff and Handley (1938) and Bloom (1950) that the undifferentiated variety of mammary carcinoma carries a worse prognosis, because of its more rapid growth, than does the more highly differentiated adenocarcinoma.

The surprisingly low figure of 16·6 per cent for prostatic carcinoma in the present series is higher than those recorded in Walther's series (11·3 per cent) and in the Montefiore series (5·9 per cent) but no obvious reasons for these discrepancies are apparent. A very high degree of histological differentiation with almost complete absence of cellular pleomorphism and mitotic activity is characteristic of many prostatic carcinomas; these features are most logically interpreted as representing a slow rate of growth and their correlation with a very marked osteosclerotic tendency is again a prominent feature in the present series. The two examples in which a significant variation in histological structure was associated with opposite bony reactions are of particular interest in this connection.

In the case of gastric carcinoma there is close agreement between the figure in the present series (10·3 per cent) and the corresponding figure (9·2 per cent) in Walther's series but the low figure (4·8 per cent) in the Montefiore series remain unexplained. There is no obvious reason for this discrepancy but the former figures are of interest since the importance of gastric carcinoma as a source of skeletal metastases was, until comparatively recently, unrecognised. Thus, Moore (1919) was unable to find a single example of sketelal metastasis in 1600 radiologically investigated cases of gastric carcinoma while Kaufmann (1929) found skeletal metastases in only 2·5 per cent of autopsies on 309 cases of gastric carcinoma. Histological analysis of the present series shows few significant features although the presence of large numbers of " signet ring " cells appears to predispose to new bone formation.

Although carcinoma of the kidney has always been considered a frequent source of skeletal metastases, low figures are recorded in the three main series of cases under discussion. This finding reflects only the low incidence of renal carcinoma in the general population since high rates of metastasis have been reported by several authors (Albrecht, 1905; Symmers, 1917; Geschickter and Copeland, 1949; Willis, 1953). All the 10 examples in the present series of cases were osteolytic, a finding with which there is general agreement. The osteolytic tendency of renal carcinomas is most easily explained by the presence of a very vascular and active stroma since the clear-celled renal carcinoma must, on histological grounds, be considered a highly differentiated adenocarcinoma.

The extremely low figures for thyroid carcinoma in the present series (1·3 per cent) and in the Montefiore series (1·8 per cent) are remarkable and again reflect the very low incidence of thyroid carcinoma in the general population, while the higher figure (6·0 per cent) reported by Walther is at least partly explained on geographical grounds (Wynder, 1952). The general and quite erroneous view prevails that carcinoma of the thyroid is a frequent source of skeletal metastases; it is in some way connected with the interest shown in the concept of "benign metastasising goitre" introduced by Cohnheim (1876) and since then shown to be fallacious by several authors (Bérard and Dunet, 1921; Bell, 1924; Simpson, 1926*b*; Schaukowski, 1956).

In conclusion several points of interest emerge from the present study. The age of the cases was in general not a significant factor although it probably explained the presence of extensive bone resorption in the skeletal metastases of a small group of well differentiated mammary adenocarcinomas. The predominance of the male sex in the present series has already been mentioned in connection with the examples of bronchial carcinoma where the usually accepted ratio of four males to one female was observed.

---

### EXPLANATION OF PLATES

FIG. 1.—Metastasis in vertebra from oat-celled bronchial carcinoma. There is apposition of new lamellar bone on the surfaces of existing trabeculae. × 60.

FIG. 2.—Metastasis in vertebra from bronchial adenocarcinoma. Extensive new bone formation has resulted from conversion of mesenchymal marrow cells into functioning osteoblasts. × 18.

FIG. 3.—Metastasis in sternum from prostatic carcinoma. Extensive necrosis has occurred in newly formed bone as shown by the disappearance of osteoblasts. × 60.

FIG. 4.—Metastasis in tibia from bronchial carcinoma. All the lacunae on the surfaces of both trabeculae are occupied by osteoclasts. × 60.

FIG. 5.—Metastasis in ilium from unknown source. The squamous-celled carcinoma has a very active fibrous stroma which is causing direct resorption of the bone trabecula. × 74.

FIG. 6.—Metastasis in rib from oat-celled bronchial carcinoma. The newly formed bone trabecula shows osteoid borders on two sides. On another side a cluster of proliferating oat cells has produced some resorption. × 250.

FIG. 7.—Metastasis in vertebra from oat-celled bronchial carcinoma. The bone shows a complete absence of reaction to the presence of the invading tumour. × 23.

FIG. 8.—Extensive new bone formation in vertebral metastasis from mammary adenocarcinoma. × 85.

FIG. 9.—Extensive new bone formation in vertebral metastasis from prostatic adenocarcinoma. × 74.

FIG. 10.—Well-differentiated metastasis in vertebra from prostatic carcinoma. Extensive new bone formation is associated with some degree of resorption. × 85.

FIG. 11.—More undifferentiated metastasis in rib from same case as shown in Fig. 10. Extensive resorption is associated with complete absence of new bone formation. × 85.

FIG. 12.—Extensive new bone formation in vertebral metastasis from "signet ring"-celled carcinoma of stomach. × 85.

1

2

3

4

5

6

7

8

Meyer.

9

10

11

12

Although malignant melanomas were excluded from the present study because of their controversial nature it is of interest that the histological files contain only one example with skeletal metastases during the period under review. This finding presumably again reflects the great rarity of this condition.

No attempt has been made to assess the frequency of pathological fracture in the series as a whole except to distinguish clearly between an osteosclerotic reaction to a tumour and new bone formation in callus resulting from a pathological fracture. The latter was, nevertheless, frequently observed in many examples of prostatic carcinoma producing extensive new bone formation in their skeletal metastases. This may be explained by consideration of the normal architecture of a bone which is adapted to provide maximum strength with minimum weight. It is clear that destruction of even small numbers of trabeculae with replacement by equal or greater numbers of irregularly arranged trabeculae must result in weakening of the bone and ultimately in pathological fracture.

### SUMMARY

A series of 320 necropsies, in which the skeletal system was involved by secondary carcinoma, has been analysed in terms of the primary site of the neoplasm and a large proportion (124 cases) was attributed to primary carcinoma of the bronchus.

Although gastric carcinoma is not generally considered a frequent source of skeletal metastases the present series included 33 examples. The very low figures for renal and thyroid carcinomas are of great interest and reflect the low incidence of these conditions in the general population.

Osteoclastic erosion was the main operative factor in resorption of bone but a direct osteolytic action was frequently exerted by the stromal cells of the tumour. In many instances resorption of bone appeared to be the result of direct pressure by masses of proliferating epithelial cells. Osteosclerosis was produced by the apposition of new bone on existing trabeculae and by the formation of new trabeculae as a result of metaplasia of marrow cells.

A complete absence of bony reaction or only a slight reaction was frequently observed in the case of the oat celled variety of bronchial carcinoma, while a relationship between osteosclerosis and a high degree of histological differentiation could be established in the case of mammary and prostatic carcinoma.

My thanks are due to my wife for help with translations and to Dr. S. Robinson for reading the paper. I am indebted to Mr. G. W. Moore for the photomicrographs and to Miss H. Pallan for secretarial work.

### REFERENCES

Abrams, H. L., Spiro, R. and Goldstein, N.—(1950) *Cancer*, **3**, 74.
Adler, I.—(1912) 'Primary Malignant Growths of the Lungs and Bronchi'. New York (Longmans, Green & Co.), 1st ed., p. 111.
Albrecht, P.—(1905) *Arch. klin. Chir.*, **77**, 1073.
Assman, H.—(1907) *Virchows Arch.*, **188**, 32.
Axhausen, G.—(1909) *Ibid.*, **195**, 358.
Barnard, W. G.—(1926) *J. Path. Bact.*, **29**, 241.
Bell, F. G.—(1924) *Brit. J. Surg.*, **12**, 331.

Bérard, L. and Dunet, C.—(1921) *Rev. Chir. Paris*, **59**, 521.
Bloom, H. J. G.—(1950) *Brit. J. Cancer*, **4**, 259.
Blumer, G.—(1909) *Johns Hopk. Hosp. Bull.*, **20**, 200.
Boyd, W.—(1953) 'A Textbook of Pathology'. London (Henry Kimpton), 6th ed., p. 622.
Bryson, C. C. and Spencer, H.—(1951) *Quart. J. Med.*, n.s., **20**, 173.
Cohnheim, J.—(1876) *Virchows Arch.*, **68**, 547.
Copeland, M. M.—(1931) *Arch. Surg. Chicago*, **23**, 581.
Doll, R. and Hill, A. B.—(1950) *Brit. med. J.*, **2**, 739.
Geschickter, C. F. and Copeland, M. M.—(1949) 'Tumors of Bone'. London, Philadelphia, Montreal (J. B. Lippincott Company), 3rd ed.
Goetsch, W.—(1906) *Beitr. path. Anat.*, **39**, 218.
Gutman, A. B. and Gutman, E. B.—(1938) *J. clin. Invest.*, **17**, 473.
Ham, A. W.—(1950) 'Histology'. Philadelphia, London, Montreal (J. B. Lippincott Company), p. 190.
Himsworth, H.—(1957) Report of M.R.C. for Year 1955–56, London (H.M.S.O.), p. 10.
Joll, C. A.—(1923) *Brit. J. Surg.*, **11**, 38.
Kaufmann, E.—(1929) 'Pathology for Students and Practitioners'. London (H. K. Lewis & Co. Ltd.), Vol. II, p. 1223.
Luck, J. V.—(1950) 'Bone and Joint Diseases'. Springfield, Illinois, U.S.A. (C. C. Thomas), 1st ed., p. 551.
Meyer, P. C.—(1956) *J. Path. Bact.*, **71**, 325.
Milch, R. A. and Changus, G. W.—(1956) *Cancer*, **9**, 340.
Moore, A. B.—(1919) *Amer. J. Roentgenol.*, n.s., **6**, 589.
Pietrogrande, V.—(1956) 'I Tumori Metastatici Dello Scheletro'. Rome (A. Staderini).
Pürckhauer, R.—(1929) *Z. Krebsforsch.*, **28**, 68.
von Recklinghausen, F.—(1891) 'Festschr. zu Virchow's 70 Geburtstage', p. 17.
Scarff, R. W. and Handley, R. S.—(1938) *Lancet*, ii, 582.
Schaukowski, R.—(1950) *Zbl. allg. Path. path. Anat.*, **86**, 19.
Schmorl, G.—(1908) *Verh. dtsch. path. Ges.*, **12**, 89.
Simpson, W. M.—(1926*a*) *Amer. J. Roentgenol.*, **15**, 534.
*Idem* (1926*b*) *Surg. Gynec. Obstet.*, **42**, 489.
Symmers, D.—(1917) *Amer. J. med. Sci.*, **154**, 225.
Walther, H. E.—(1948) 'Krebsmetastasen'. Basel. (Benno Schwabe & Co.).
Weese, K.—(1956) *Virchows Arch.*, **329**, 1.
Willis, R. A.—(1952) 'The Spread of Tumours in the Human Body'. London (Butterworth & Co.), 2nd ed., p. 242.
*Idem* (1953) 'Pathology of Tumours'. London (Butterworth & Co.), 2nd ed.
Wynder, E. L.—(1952) *New Engl. J. Med.*, **246**, 573.
Zemgulys, J.—(1931) *Z. Krebsforsch.*, **34**, 266.